# Evaluating the Incidence of Incidental Gallbladder Carcinoma in a Tertiary Care Centre: A Retrospective Analysis in North India

**DOI:** 10.7759/cureus.76217

**Published:** 2024-12-22

**Authors:** Shalini Bhalla, Nida Shabbir, Kusum Yadav, Manish Kumar, Nidhi Gupta, Sneha Chaudhary, Mithilesh, Akanksha Sharma, Preeti Agarwal

**Affiliations:** 1 Pathology, King George's Medical University, Lucknow, IND; 2 Pathology, Dr. Ram Manohar Lohia Institute of Medical Sciences, Lucknow, IND; 3 Pathology, Dr. KNS Memorial Institute of Medical Sciences, Lucknow, IND; 4 Pathology, Rama Medical College, Hospital and Research Centre, Hapur, IND

**Keywords:** cholecystectomy, early detection, histopathology, incidental gallbladder cancer, tertiary care

## Abstract

Background

Incidental gallbladder carcinoma (IGBC) remains a significant clinical challenge, with its diagnosis often delayed due to the asymptomatic nature of the disease and its incidental discovery post-cholecystectomy. This study's aim is to calculate incidence in a high-risk, region-specific (North Indian) population and also to provide novel insights into clinical presentation as well as macroscopic and histopathological features of IGBC.

Material and methods

This retrospective observational study spanned four years (August 2013 to July 2016) and included a total of 3096 cases. Demographic, clinical, radiological, treatment and follow-up data were sourced from archived records. IGBC diagnoses were confirmed on formalin-fixed, paraffin-embedded tissue sections stained with hematoxylin and eosin (H&E).

Results

A total of 3,067 routine cholecystectomies were performed during this period, of which a total of 162 cases (18.93%) were diagnosed as gallbladder carcinoma (GBC) and 51 cases (1.74%) were identified as IGBC. The mean age was 49.8 years. Among the total IGBC cases, most patients underwent preoperative imaging of which eight cases (15.6%) showed findings on preoperative radiological evaluation suggestive of malignancy. Among these, five cases (9.8%) demonstrated subtle gallbladder wall thickening and three cases (5.8%) revealed suspicious small polypoidal lesions (less than 1 cm). Microscopy showed that majority of these tumours were well-differentiated adenocarcinomas (n=27; 52.94%), followed by moderately differentiated adenocarcinomas (n=17; 33.33%), mucinous adenocarcinomas (n=03; 5.88%), papillary adenocarcinomas (n=02; 3.92%), and neuroendocrine tumours (n=1; 1.96%). Staging revealed 26 (50.98%) cases as Stage IIA, 24 (47.0%) as Stage I, and one (1.9%) as Stage III. On follow-up, the median survival period was found to be 32 months.

Conclusions

Our study emphasizes the limitations of conventional imaging in detecting early-stage gallbladder cancer, and it advocated the critical importance of routine histopathological examination (HPE) of all gallbladder specimens. Additionally, our findings contribute to a growing body of evidence that suggests early-stage IGBC may offer improved survival outcomes if diagnosed timely and treated aggressively, prompting a re-evaluation of current diagnostic and management strategies.

## Introduction

Gallbladder carcinoma (GBC) is the most common malignancy of the biliary system and ranks as the fifth most prevalent gastrointestinal cancer [1]. Incidental gallbladder carcinoma (IGBC) refers to cases where GBC is diagnosed only through histopathological examination of cholecystectomy specimens, without preoperative suspicion from imaging methods such as ultrasound or CT scan [2]. Globally, IGBC detection rates vary but remain a particular concern in regions with high rates of gallbladder disease. In India, for instance, the incidence of gallbladder carcinoma showed marked geographic variability, with significantly higher rates in northern regions, including Delhi, than in the southern part of the country [3].

IGBC poses a substantial clinical challenge due to its typically asymptomatic presentation and lack of preoperative suspicion, underscoring the importance of histopathological evaluation for an accurate diagnosis [4]. The pathogenesis of GBC is multifactorial, with chronic inflammation due to gallstones as a primary risk factor [5]. Other risk factors include female gender, older age, and certain ethnic or regional predispositions [6]. The incidental nature of IGBC highlights the limitations of current imaging techniques for preoperative detection, as ultrasound (USG) and computed tomography (CT) often miss early or small lesions. In contrast, preoperative diagnoses are generally associated with advanced tumors that have already invaded adjacent structures [7]. Notably, IGBC tends to have a more favorable prognosis than symptomatic GBC, often due to its detection at an earlier stage as compared to GBC which is commonly detected at a higher stage [8,9]. For patients with locally advanced or metastatic GBC, treatment with gemcitabine and cisplatin remains the standard, based on survival benefits demonstrated in the ABC-02 trial, although prognosis remains poor with median survival under one year [10]. Among those eligible for curative resection, adjuvant chemotherapy has shown improved outcomes, with the BILCAP trial indicating that adjuvant capecitabine extends overall survival by 17 months in surgically resected biliary tract cancer patients, including GBC cases [11].

This retrospective study investigates the incidence of IGBC among patients undergoing cholecystectomy at a tertiary care center and contributes to improved clinical management of GBC. With a large sample size and detailed analysis, the study aims to not only assess IGBC incidence but also to explore diagnostic limitations and clinical gaps in preoperative assessment. Additionally, it evaluates the potential of routine histopathological examination to enhance early detection rates and prognosis. By providing an in-depth characterization of IGBC and comparing it with current diagnostic standards, this study offers valuable insights for optimizing clinical outcomes in gallbladder cancer.

## Materials and methods

This retrospective, observational study was conducted at the Department of Pathology, King George's Medical University, Lucknow, a tertiary care center in North India. Medical records of all patients who underwent cholecystectomy from August 2013 to July 2016 were reviewed, with an examination of histopathology reports to identify cases of IGBC. Inclusion criteria were patients who had elective or emergency cholecystectomy for suspected benign gallbladder conditions, such as cholelithiasis or cholecystitis, and a postoperative IGBC diagnosis confirmed by histopathology. Patients with a preoperative diagnosis or strong clinical suspicion of gallbladder cancer, or those without histopathological analysis, were excluded.

IGBC diagnosis was confirmed using formalin-fixed, paraffin-embedded tissue sections stained with H&E (hematoxylin & eosin) stain. Cancer staging followed the American Joint Committee on Cancer (AJCC) guidelines, 2008 [12]. Gallbladder wall thickness was categorized as abnormal if exceeding 3 mm on preoperative imaging or histopathological examination, with normal thickness typically between 1-2 mm.

Data were retrospectively collected from 2013 to 2016, including demographics, clinical presentations before surgery (e.g., pain, jaundice, fever, gallstone presence), preoperative imaging findings (USG, CT, MRI), histopathological details (cancer stage and grade), and postoperative management (e.g., re-resection, chemotherapy, or radiotherapy following diagnosis).

All data collected were compiled in Microsoft Excel (Microsoft Corporation, Redmond, WA), and statistical analyses were conducted using IBM SPSS Statistics for Windows, Version 27 (IBM Corp., Armonk, NY).

## Results

Demographic findings and incidence

**Figure 1:**
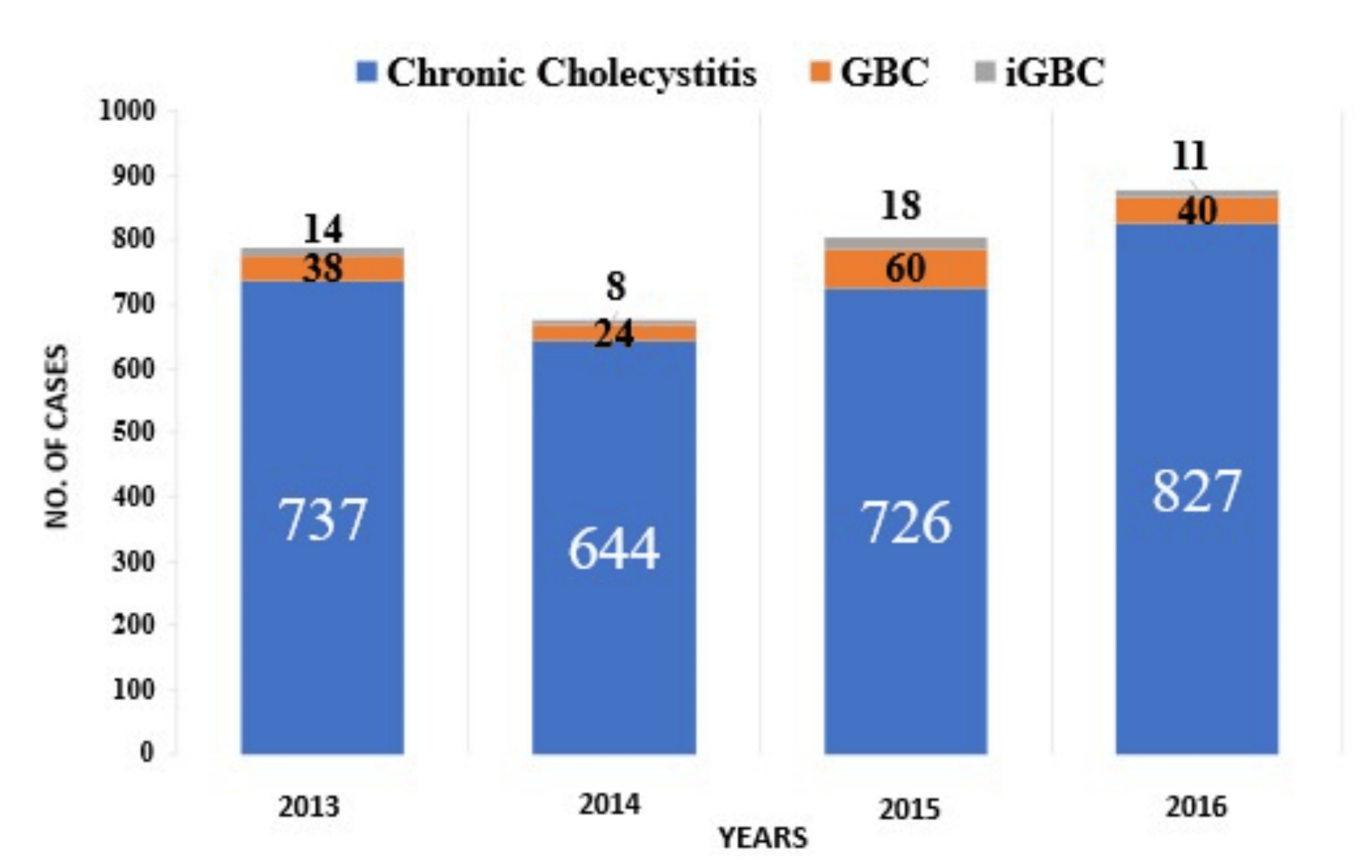
Histogram of showing distribution of chronic cholecystitis, GBC and IGBC GBC: gallbladder carcinoma, IGBC: incidental gallbladder carcinoma

A total of 3,067 cholecystectomy specimens were received by the Department of Histopathology at King Georges Medical University over a four-year period (2013-2016). These specimens, obtained from both open and laparoscopic cholecystectomies, as well as radical cholecystectomies, were primarily collected for the management of clinically and radiologically diagnosed benign gallbladder diseases. The mean age of patients in the study showed a general upward trend over the years, beginning at 48.78 years in 2013, briefly decreasing to 42.62 in 2014, then increasing to 52.11 in 2015 and 55.90 years in 2016. The combined mean age over the four-year period was 49.8 years. Female patients consistently outnumbered male patients each year, with a male-to-female (M:F) ratio of 2:15. Most cholecystectomy specimens were diagnosed as chronic cholecystitis (Figure 1; Table 1).

**Table 1:** Yearwise frequency distribution of demographic characteristics of cases enrolled.

Variables	2013	2014	2015	2016
Total cholecystectomy	775	668	786	838
Chronic cholecystitis	737	644	726	827
Cumulative mean age (years)	49.85			
Gender (M:F ratio)	01:13	1:1.6	1:8	00:11
GBC	38 (4.9%)	24 (3.5%)	60 (7.6%)	40 (4.7%)
Mean age of IGBC patients (years)	48.78	42.62	52.11	55.9
IGBC	14 (1.8%)	8 (1.19%)	18 (2.2%)	11 (1.3%)
Incidence IGBC (yearly)	1.89%	1.24%	2.47%	1.33%
Cumulative incidence IGBC (4 years, 2013-2016)	1.74%			

**Figure 2:**
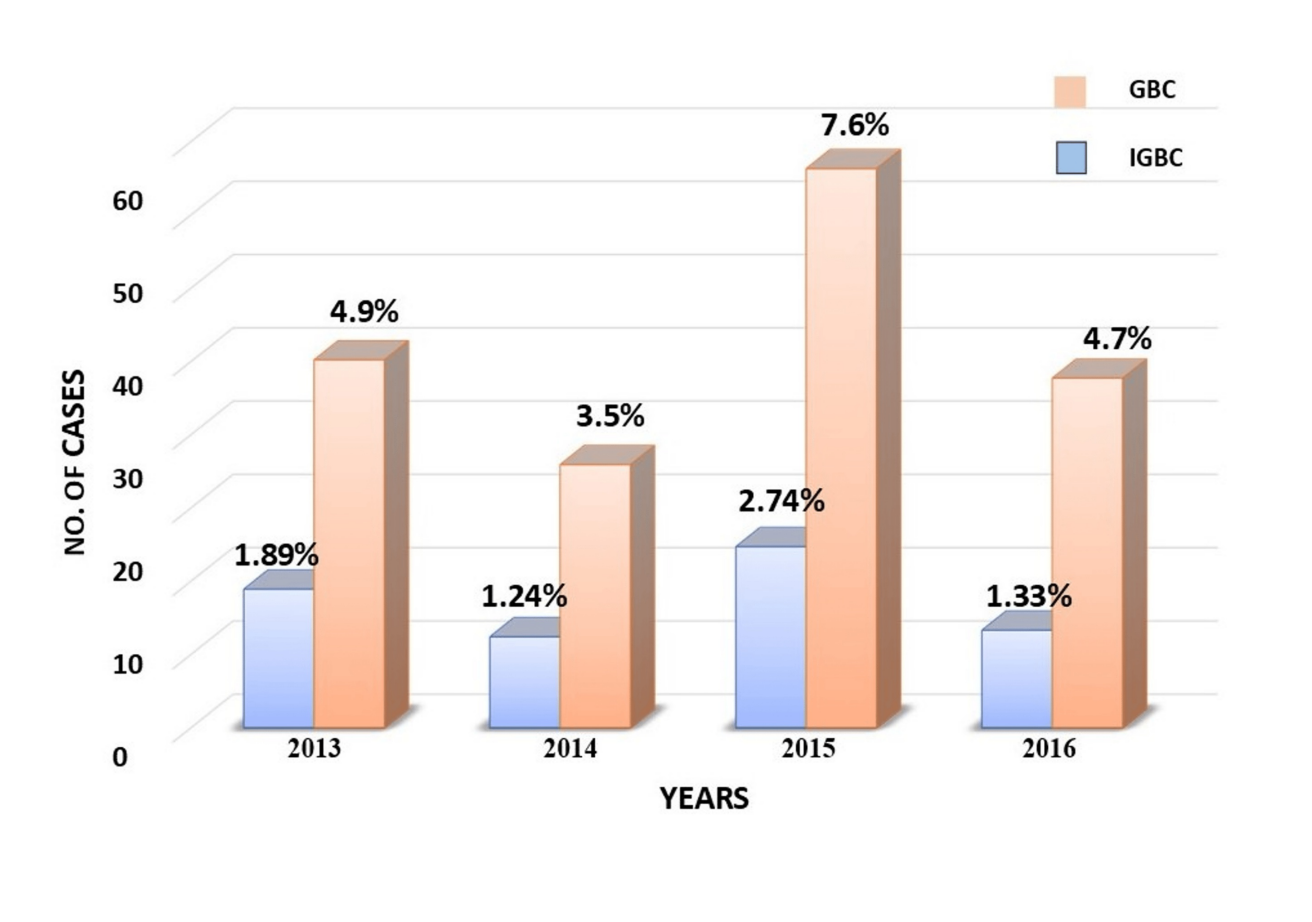
3-D histogram of showing distribution of GBC and IGBC GBC: gallbladder carcinoma, IGBC: incidental gallbladder carcinoma

GBC was identified in 162 specimens (5.23%), with a notable peak incidence in 2015, where 60 (37.03%) cases were reported, yielding an incidence rate of 7.63% for that year. The occurrence of IGBC, typically detected post-cholecystectomy for benign conditions like chronic cholecystitis, varied over the study period. Upon microscopic examination, IGBC was identified in 51 cases (1.6% of total cases), with diagnoses made histopathologically without prior clinical or radiological suspicion of malignancy (Table 2). The annual incidence rates of IGBC were 1.89% in 2013, and 1.24% in 2014, reaching a peak of 2.47% in 2015, and subsequently decreasing to 1.33% in 2016, with an overall cumulative incidence of 1.74% (Figure 2).

**Table 2:** Yearwise frequency distribution of tumour characteristics of GBC and IGBC. GBC: gallbladder carcinoma, IGBC: incidental gallbladder carcinoma

Variables	Years			
	2013	2014	2015	2016
Specimen received				
Biopsy	2 (5.2%)	3 (12.5%)	9 (15%)	6 (15%)
Resected GB	35 (92%)	19 (79.1%)	36(60%)	14 (35%)
GB with part of liver	3 (7.8%)	2 (8.3%)	15(25%)	18 (45%)
Specimen sent from				
Our institute	34 (89.4%)	23 (95.8%)	6 (100%)	37 (92.5%)
From outside	4 (10.5%)	1 (4.1%)	0 (0%)	3 (7.5%)
Tumor size				
<1 cm	17 (44.7%)	17 (70.8%)	37 (61.6%)	12 (30%)
1-2 cm	7 (18.4%)	3 (12.5%)	8 (13.3%)	10 (25%)
2-5 cm	10 (26.3%)	3 (12.5%)	11 (18.3%)	13 (32.5%)
> 5 cm	4 (10.5%)	1 (4.1%)	4 (6.6%)	5 (12.5%)
Tumor location				
Neck	3 (7.8%)	3 (12.5%)	5 (8.3%)	5 (12.5%)
Body	12 (31.5%)	12 (50%)	37 (61.6%)	19 (47.5%)
Fundus	22 (57.8%)	8 (33.3%)	14 (23.3%)	15 (37.5%)
Diffuse	1 (2.6%)	1 (4.1%)	4 (66.6%)	1 (2.5%)
Microscopic tumour extension				
Up to muscle layer	3 (7.8%)	3 (12.5%)	15 (25%)	7 (17.5%)
Up to serosa	32 (84.2%)	18 (75%)	42 (70%)	33 (82.5%)
Infiltrating liver	2 (5.2%)	2 (8.3%)	13 (21.6%)	10 (25%)
Distant metastasis	1(2.6%)	1 (4.1%)	0 (0%)	0 (0%)
Histological variants				
Well-differentiated	14 (36.8%)	7 (29.1%)	22 (36.6%)	16 (15%)
Moderately differentiated	19 (50%)	13 (54.1%)	32 (53.3%)	20 (50%)
Poorly differentiated	0(0%)	0(0%)	2 (3.3%)	1 (2.5%)
Papillary adenocarcinoma	3 (7.8%)	0(0%)	1(1.6%)	1(2.5%)
Mucinous adenocarcinoma	2 (5.2%)	1 (4.1%)	1(1.6%)	1(2.5%)
Others	0(0%)	3 (7.8%)	2 (3.3%)	1 (2.5%)
Positive lymph nodes				
1-2	3 (7.8%)	3 (12.5%)	6 (10%)	2 (5%)
2-5 LN	0 (0%)	1 (4.1%)	0 (0%)	2 (5%)
5-10 LN	1 (2.6%)	0 (0%)	1 (1.6%)	1 (2.5%)
>10 LN	0 (0%)	0 (0%)	0 (0%)	0 (0%)
Pathological staging (AJCC 2018, 8th edition)				
pT1N0Mx	6 (15.7%)	2 (5.2%)	10 (16.6%)	6 (15%)
pT1N1Mx	0 (0%)	2 (8.3%)	1 (1.6%)	1 (2.5%)
pT2N0Mx	15 (39.4%)	13 (54.1%)	20 (33.2%)	9 (22.5%)
pT2N1Mx	4 (10.5%)	1 (4.1%)	4 (6.6%)	3 (7.5%)
pT2N2Mx	0 (0%)	0 (0%)	0 (0%)	0 (0%)
pT3N0Mx	6 (15.7%)	2 (8.3%)	11 (18.3%)	8 (20%)
pT3N1Mx	1 (2.6%)	1 (4.1%)	4 (6.6%)	1 (2.5%)

Shortfalls in preoperative imaging and diagnosis

**Figure 3:**
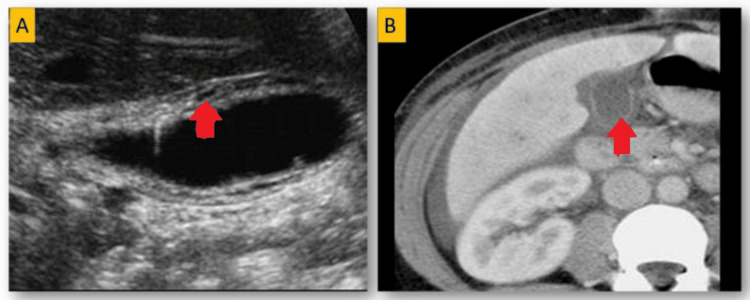
Radiology pictures of IGBC A: Digital ultrasonography picture of IGBC showing thickened gallbladder wall. B: CT image of IGBC showing thickened gallbladder wall. Abbrevations: IGBC=incidental gallbladder carcinoma

Among the total IGBC cases, most patients underwent preoperative imaging, including ultrasound (USG), computed tomography (CT), or magnetic resonance imaging (MRI), as part of their diagnostic workup. However, only a small subset, eight cases (15.6%) showed findings on preoperative radiological evaluation suggestive of malignancy. Among these, five cases (9.8%) demonstrated subtle gallbladder wall thickening of approximately 3-5 mm, which was initially considered non-specific (Figure 3A-3B).

Three cases (5.8%) revealed suspicious, small polypoidal lesions (less than 1 cm) within the gallbladder, raising mild concern for possible malignancy. The majority of IGBC cases (84.4%) appeared radiologically unremarkable.

Divergent macroscopic profiles

**Figure 4:**
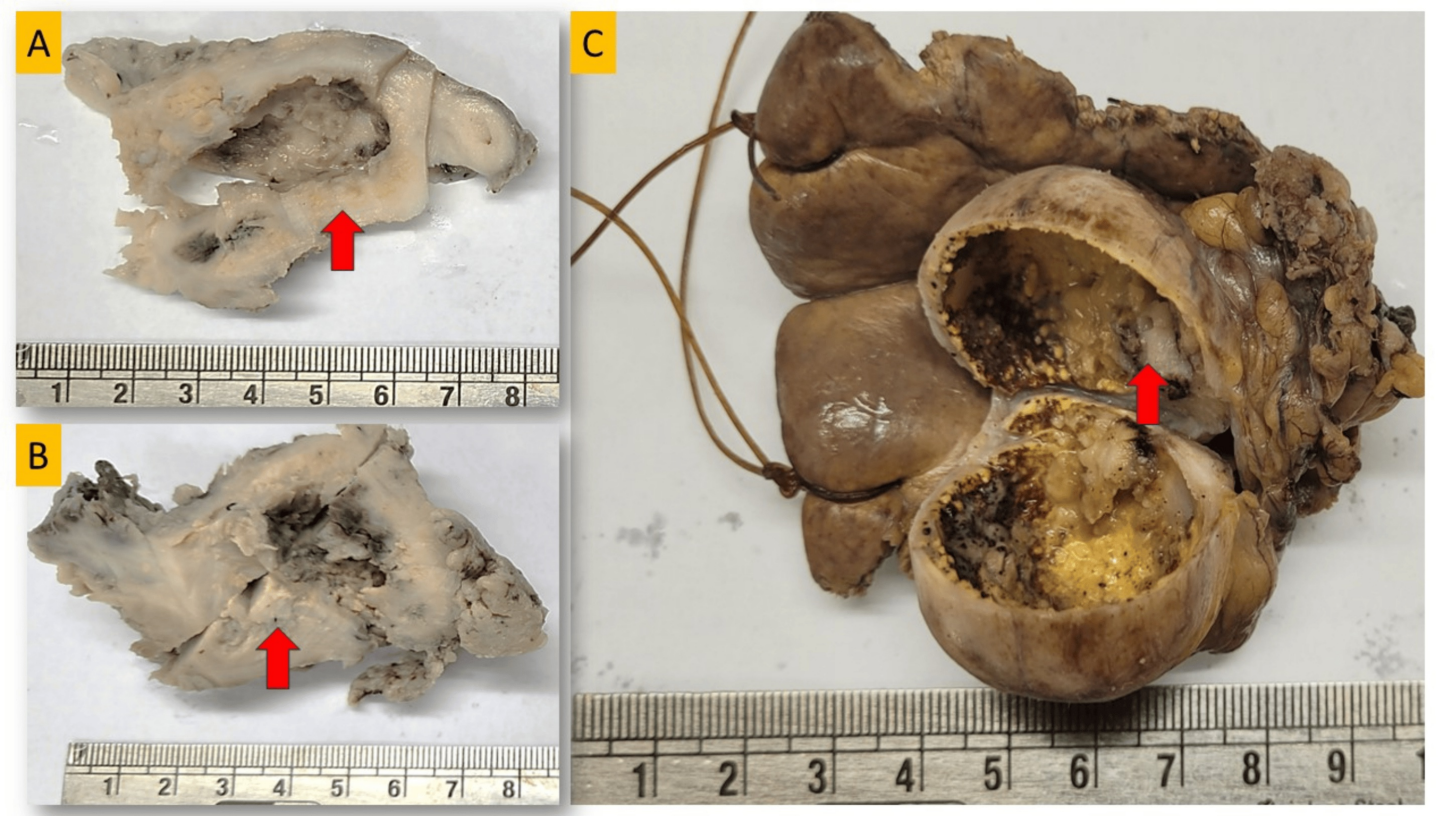
Gross pictures of enrolled cases reported as IGBC A: Gross picture showing papillary projections, reported as IGBC in microscopy B: Gross picture showing thick wall with yellow-colored streaks with papillary projections in lumen reported as IGBC with xanthogranulomatous chronic cholecystitis in microscopy. C: Gross picture with arrow showing grayish-white solid growth in GB lumen and multiple yellow/black colored stones. Abbreviations: GB=gallbladder, IGBC=incidental gallbladder carcinoma

In the present study of 51 IGBC cases, only 14 (27.4%) exhibited notable macroscopic abnormalities. These included diffusely thickened gallbladder walls (>3 mm) in four cases (7.8%), polypoidal growths in eight cases (15.6%), and papillary excrescences in two cases (3.9%). Cases also showed yellowish fatty streaks with papillary excrescences (Figure 4A-4C), further diagnosed as adenocarcinoma NOS with associated xanthogranulomatous cholecystitis. These lesions were primarily located in the body of the gallbladder (n=16, 31.3%), followed by the fundus (n=14, 27.4%) and neck (n=5, 9.8%). The remaining specimens appeared grossly unremarkable. A significant difference in gallbladder wall thickness was noted between pre-diagnosed tumors and incidental cases, with diagnosed tumors showing prominent thickening (>6 mm) compared to IGBC cases, which often showed minimal (>3 mm) or no thickening. This contrast underscores the subtle macroscopic features that can complicate early detection of IGBC.

Histopathological analysis

**Figure 5:**
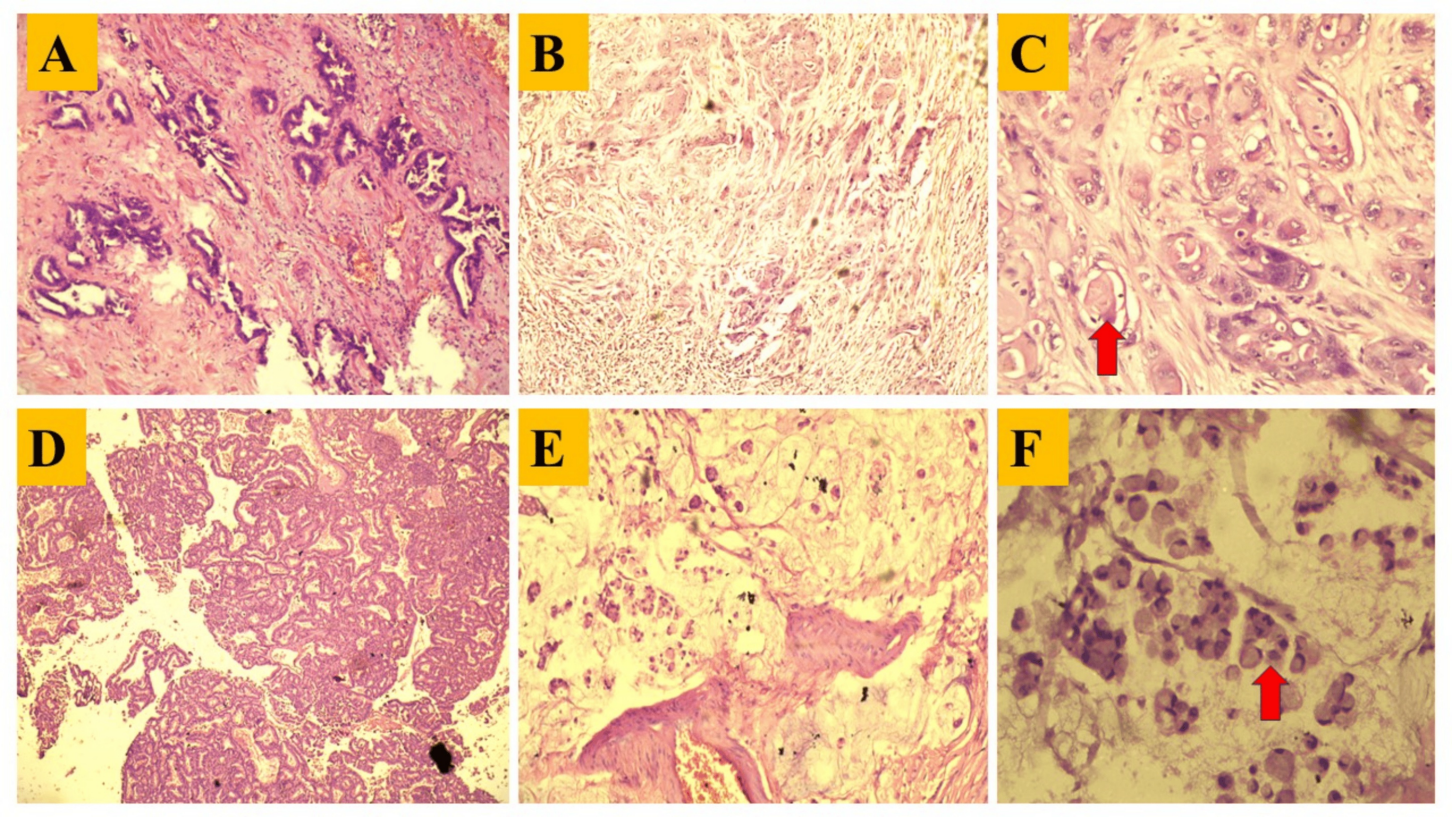
Photomicrographs of IGBC depicting various histological variants A: Photomicrograph showing well differentiated adenocarcinoma. B: Photomicrograph showing adenosquamous carcinoma in low power view (100X). C: Photomicrograph showing adenosquamous carcinoma in High power (400X). Arrow showing keratin pearl formation. D: Photomicrograph showing intracholecystic papillary neoplasm (ICPN). E: Photomicrograph showing mucinous adenocarcinoma in low power view (100X). F: Photomicrograph showing mucinous adenocarcinoma in high power view (400X). Arrow showing signet ring cell. Abbreviation: IGBC=incidental gallbladder carcinoma

**Figure 6:**
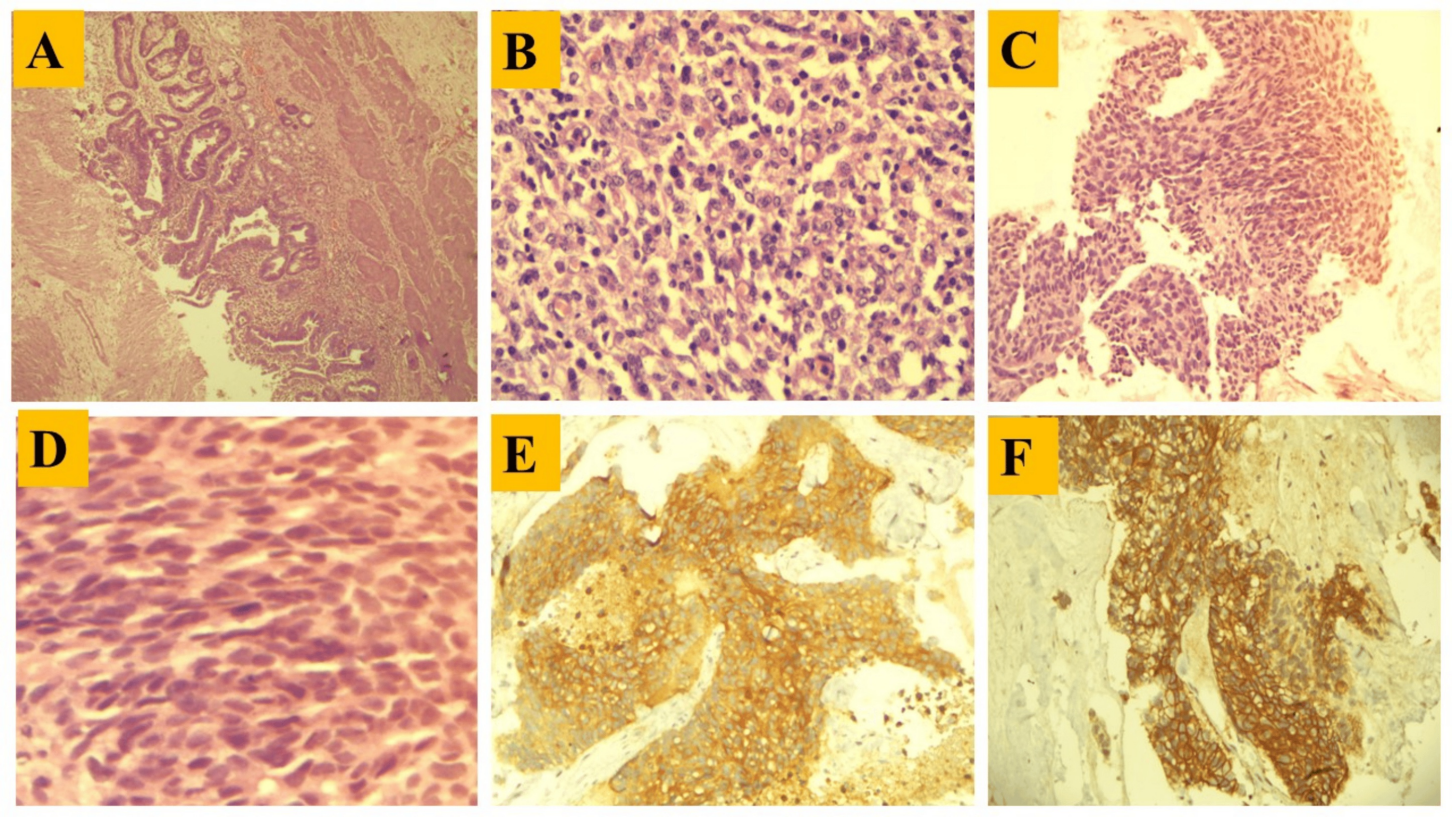
Photomicrograph of IGBC histology and immunohistochemistry. A: Photomicrograph showing papillary adenocarcinoma in low power view (100X). B: Photomicrograph showing poorly differentiated adenocarcinoma. C: Photomicrograph showing small cell carcinoma in low power view (100X). D: Photomicrograph showing small cell carcinoma in High power view (400X). E: Photomicrograph showing positive synaptophysin immunohistochemistry marker in small cell carcinoma. F: Photomicrograph showing positive CD56 immunohistochemistry marker in small cell carcinoma. Abbreviation: IGBC=incidental gallbladder carcinoma

Most specimens were obtained from within our institution, with a smaller number referred from external centers (government and private hospitals). Histological analysis identified adenocarcinoma NOS as the predominant tumor subtype. Further subclassification revealed that the majority of these tumors were well-differentiated adenocarcinomas (n=27; 52.94%), (Figure 5A) consistently observed throughout the study period. This was followed by moderately differentiated adenocarcinomas (n=17; 33.33%), mucinous adenocarcinomas (n=03; 5.88%), papillary adenocarcinomas (n=02; 3.92%), and neuroendocrine tumors (n=1; 1.96%). Adenosquamous cell carcinoma was rare, with only a single case (1.9%) documented (Figure 5B-5F; Figure 6A-6B). Cases of neuroendocrine carcinoma (small cell carcinoma) were also identified on histology and further came out to be positive for synaptophysin and CD56 IHC (Figure 6C-6F).

Cystic duct margin was involved in only three cases. Examination of adjacent structures showed occasional direct extension into the liver (n=2, 3.9%). Most patients were staged at T2 (n=31, 60.7%), followed by T1 (n=18, 35.2%). Nodal metastasis was observed in both T1 (n=3, 5.8%) and T2 (n=3, 5.8%) stages. Based on the 2018 cancer staging guidelines of the American Joint Committee on Cancer (AJCC 8th edition), of total GBC cases (n=162) reported, the majority of patients (n=57, 35.18%) were classified as Stage IIA, followed by Stage Iii (46, 28.3%), with Stage I (24, 14.81%) presenting infrequently in isolated cases across the years. IGBC diagnosed cases (n=51) staging revealed 26( 50.98%) cases as Stage IIA, 24 (47.0%) cases as Stage I, and one (1.9%) case as Stage III.

Survival and postoperative outcomes

During follow-up, the median survival period was found to be 32 months. In our study, 13 (25.4%) patients with pT1b and pT2 stages of IGBC underwent further radical resection. Among them, patients (N=8,61.5%) with early-stage IGBC (pT1b) showed significantly better outcomes following chemotherapy or radiotherapy compared to those with advanced stages (pT2). The remaining (N=5,38.4%) patients succumbed to the disease, with a median survival of 12 months. These findings emphasize the curative potential of radical resection in early-stage IGBC and highlight the vital role of histopathological examination (HPE) in guiding postoperative management strategies.

## Discussion

We included cholecystectomy cases from both open and laparoscopic surgical approaches as the survival rate of IGBC cases was statistically correlated with tumor stage but not with the type of surgical approach used [13]. The mean age of IGBC patients in our study was 49.85 years, with a gradual increase over the study period, 48.78 years in 2013, 42.62 in 2014, 52.11 in 2015, and 55.90 in 2016. This average age is relatively lower than that reported in other studies, such as 58.6 years by Yadav et al. [14] (Table 3), 63.8 years by Alabi et al., [15] and 65 years by Ghimire et al. [16]. However, it closely aligns with findings from previous studies by Jha et al. [17] and Poudel et al. (49.14 and 50.6 years, respectively) [18]. The increasing mean age of IGBC patients may reflect improved diagnostics, greater incidental findings in older adults, longer exposure to risk factors, and a higher prevalence of obesity and gallstones. Cohort-specific risks and delayed healthcare-seeking in older adults may also play a role.

Gender distribution notably showed a predominance of female patients across all years (88.2%), aligning with previous studies by Yadav et al. [14], Jha et al. [17], Poudel et al. [18] and Omhare et al. [19] and reflecting global epidemiological trends of female predominance. This is likely attributable to the role of estrogen in increasing bile cholesterol saturation, thereby elevating the risk of gallstone formation.

The cumulative incidence of IGBC in our study (1.74%) aligns with previous trials and meta-analyses, reported IGBC incidence range of 0.19% to 2.8%. Our incidence falls within this range and closely matches findings by Poudel et al. [18], Omhare [19] et al., and Sangwan et al. [20] with incidences of 1.67%, 1.82%, and 1.9%, respectively In contrast, lower incidences were observed in studies by Jha et al. [17], and Gulwani et al. [21], (0.41%, 0.76%, respectively) (Table 3).

**Table 3:** Comparison of incidence, pre-operative radiological and macroscopic findings of IGBC IGBC: incidental gallbladder carcinoma

Studies	Sample size	No. of cases	Incidence of IGBC	M: F	Mean Age	Preoperative radiological findings	Macroscopic findings
Present study	3096	51	1.74%.	1:7.5	49.8 years	Thickened wall (n=5), polypoidal mass (n=3)	Thickened wall, papillary excrescences, and polypoidal mass
Yadav et al. [14]	1268	16	1.26%	1:7	58.63 years	All cases diagnosed as chronic cholecystitis and cholelithiasis	Thickened wall in all cases
Alabi et al. [15]	1473	2	0.14%	Female patients only (n=2)	65	Acute cholecystitis/ gallstone pancreatitis	Thickened wall
Ghimire et al. [16]	783	10	1.28%	1:2.3	63.8 years	Unremarkable findings	Polypoidal mass (n=2), thickened wall (n=1), cases with no gross abnormality (n=7)
Jha et al. [17]	4800	20	0.41%	1:2.3	50.65 years	Thickened wall (n=6)	Thickened wall (n=11), ulceration (n=2)
Poudel et al. [18]	418	7	1.67%	6:1	49.14	No suspicion of malignancy	No findings
Omhare et al. [19]	383	7	1.82%	1:6	46.4	Cholelithiasis	Gallstones only
Sangwan et al. [20]	530	10	1.9%	6:1	44.16	Cholelithiasis	Gallstones and thickened wall
Gulwani et al. [21]	2990	23	0.76 %	1:1.9	57.8 years	Fine papillary (n=1), fleshy mass (n=1), mesenteric lymphadenopathy (n=1)	Wall thickness (n=21), papillary (n=1), polypoidal mass (n=1)

Annual evaluations revealed fluctuations in IGBC incidence over the study period, with rates of 1.89%, 1.24%, 2.47%, and 1.33% for the years 2013, 2014, 2015, and 2016, respectively. The spike observed in 2015 may suggest improvements in diagnostic practices or increased awareness, with the higher detection of IGBC potentially reflecting more comprehensive histopathological examinations following cholecystectomy. These trends may indicate evolving surgical and pathological protocols or shifts in regional gallbladder cancer incidence. This underscores the critical importance of routine histopathological examination of all gallbladder specimens, even when there is no preoperative suspicion of malignancy.

Among 51 IGBC cases, only a small subset of eight cases (15.6%) showed radiological findings suggestive of malignancy. Of these, five (9.8%) cases demonstrated subtle gallbladder wall thickening (3-5 mm), consistent with findings from Jha et al. Three (5.8%) cases had small, suspicious polypoidal lesions (<1 cm), though these could be mistaken for benign conditions like chronic cholecystitis. The majority of IGBC cases (84.4%) appeared radiologically unremarkable, underscoring the limitations of imaging in detecting early or incidental IGBC and highlighting the need for histopathological examination for accurate diagnosis. Our study found that most IGBC cases were linked to cholelithiasis, consistent with findings by Yadav et al. [14] and Gulwani et al., [21] supporting the theory that chronic inflammation from gallstones plays a key role in gallbladder carcinogenesis. However, our results suggest that advanced age, especially in postmenopausal women, could be a more critical factor than gender alone in the development of IGBC. Our study highlights the limitations of current preoperative imaging in accurately diagnosing early-stage gallbladder cancer. The low sensitivity of standard imaging reveals a critical diagnostic gap, emphasizing the need for innovative approaches, such as liquid biopsies or tumor-specific molecular markers, to enhance early detection [22]. Refining radiological criteria for IGBC could help differentiate benign from potentially malignant conditions, improving preoperative identification. Most cases were found postoperatively, often missing the chance for curative intervention. Establishing intraoperative frozen section protocols based on suspicious features could enable more timely interventions.

Histologically, the predominant tumor subtype in IGBC was well-differentiated (WD) adenocarcinoma (52.94%), followed by moderately differentiated (MD) adenocarcinoma (33.33%), mucinous adenocarcinoma (5.88%), papillary adenocarcinoma (3.92%), and neuroendocrine tumor (1.96%). Squamous cell carcinoma was rare, identified in only one case. Adenocarcinomas (NOS) is the most common histological subtype in malignant gallbladder neoplasms, accounting for 90-95% of all cases, consistent with this, our study also showed a high occurrence of adenocarcinoma in IGBC cases. In the study by Ghimire et al., most cases were adenocarcinoma, with two adeno-squamous carcinoma cases. Omhare et al. reported all cases as adenocarcinoma with an equal distribution of WD and MD types while Jha et al. [17] and Gulwani et al., [21] found that most cases were MD adenocarcinoma. In our study, papillary adenocarcinoma was found in (3.92%) of cases, while the study by Gulwani had an incidence rate of 16.2% [21]. The detailed histopathological analysis presented here, particularly the identification of rarer subtypes of gallbladder cancer, adds a layer of complexity to understanding IGBC. Different histological types may respond uniquely to treatment, and early recognition of these distinctions can guide tailored therapies, potentially improving outcomes for specific patient groups. Cystic duct margin was involved in only three (5.8%) cases in our study in comparison to Gulwani et al (n=4) [21].

 In this study most patients were in the early stages, with T2 (n=31,60.7%) being the most common, followed by T1 (n=18,35.29%). Nodal metastasis was observed in both T1 (n=3,5.8%) and T2 (n=3,5.8%) stages, aligning with findings from Poudel et al., Jha et al. [17], and Gulwani et al. [21]. In our study, only two patients were T3, whereas Yadav et al. [14] reported five cases at the T3 stage. Pathological staging based on AJCC 2018 guidelines revealed that most patients were at Stage II, followed by Stage I, while the less common Stage III was observed only in one case, consistent with previous studies. These early-stage cancers are often undetectable on imaging, reinforcing the critical role of routine histopathological examination (HPE) in diagnosing malignancies that might otherwise progress undetected until symptomatic stages.

Recent studies indicate that up to 70% of IGBC cases initially exhibit imaging features similar to benign conditions like chronic cholecystitis or cholelithiasis. This lack of specificity highlights a significant gap in clinical practice, suggesting that advanced imaging modalities, such as contrast-enhanced ultrasound (CEUS) and diffusion-weighted MRI, along with emerging molecular markers, could enhance early malignancy detection rates [23]. Additionally, AI-driven image analysis shows promise in improving sensitivity by identifying subtle tissue changes associated with malignancy, potentially enabling earlier and more accurate diagnoses of IGBC [24]. In our study, 14 (27.4%) cases show notable microscopic findings with diffusely thickened gallbladder walls (>3 mm) in four cases (28.5%), polypoidal growths in eight cases (57.1%), and papillary excrescences in two cases (14.2%), which were later confirmed microscopically, consistent with previous studies [25]. In contrast, a study by Talreja et al. reported macroscopic abnormalities in all IGBC patients [26]. Hence, our study emphasized the importance of HPE for gallbladder specimens and noted that it is necessary to examine the specimens even in the absence of clinical or macroscopical suspicion.

The treatment and prognosis of gallbladder cancer largely depend on the tumor stage. For Tis and pT1a tumors, a simple cholecystectomy is sufficient, offering a nearly 100% five-year survival rate. However, for pT1b tumors or more advanced stages, radical re-resection is recommended. In pT2 tumors, the five-year survival rate significantly improves from 20% to 70% when a simple cholecystectomy is followed by radical cholecystectomy upon IGBC diagnosis [27]. In our study, 13 (64.70%) patients of pT1b and pT2 underwent further radical resection, aligning with findings by Jha et al. [17] and Glauser et al. [27]. On follow-up, four (7.8%) out of eight patients were alive and disease-free at the final follow-up (median 32 months). The rest of the patients with GBC died of the disease, with a median survival of 12 months. Current guidelines recommend that routine histopathological analysis of cholecystectomy specimens be performed to enable early detection and improve survival outcomes for gallbladder cancer patients [28,29]. While studies indicate that simple cholecystectomy is effective for pT1a IGBC, offering a five-year survival rate of 100%, further radical resection is advised for pT1b tumors to enhance prognosis. Our study showed that most IGBC cases were identified at the pT2 stage, where survival rates can increase significantly if simple cholecystectomy is followed by radical resection [30,31]. Additionally, Frena et al. reported that extended cholecystectomy can raise survival rates to 92-100% [32].

Our findings indicate that early-stage IGBC patients, especially those diagnosed at stage I or II, have significantly improved survival rates. This suggests that enhanced diagnostic tools and a more proactive surgical approach to suspicious gallbladder pathology could greatly improve survival. Future studies should investigate the role of advanced imaging, molecular markers, and genetic testing to enhance preoperative detection of IGBC. Additionally, clinical guidelines on IGBC management, including re-resection and adjuvant therapies, could further support better patient outcomes.

Strengths and limitations of the study

This study examines the incidence of IGBC in a large North Indian population and presents several strengths. Notably, the large sample size enhances the statistical power and provides a more robust representation of the regional incidence, offering valuable insights into IGBC occurrence. Further detailed study of tumor characteristics on histopathology provides critical information about IGCB and GCB. Additionally, the focus on preoperative diagnostic strategies addresses a critical area in gallbladder cancer management, underscoring the need for improved detection methods for earlier and more accurate diagnosis of IGBC cases.

However, there are several limitations. As the study was retrospective, it relies on existing records, which may lead to incomplete data and potential selection bias. Additionally, while it highlights the need for enhanced preoperative diagnostics, the study does not implement or test specific diagnostic improvements, limiting its direct applicability to clinical practice. Further prospective studies focusing on advanced imaging or molecular markers are warranted to validate these findings and support enhancements in preoperative screening.

## Conclusions

This retrospective study reveals key insights into the incidence and characteristics of IGBC in a tertiary care setting. It underscores the limitations of current imaging for early detection and stresses the essential role of routine HPE to identify undiagnosed IGBC cases. Early-stage diagnosis, especially in Stage I or II, significantly improves survival, highlighting the need for improved preoperative diagnostics and advanced imaging tools. In a region with a high prevalence of gallbladder cancer, routine HPE of cholecystectomy specimens is essential to ensure early detection and reduce disease burden. Improving diagnostic strategies will be crucial in enhancing survival rates and advancing gallbladder cancer management.
